# Correction

**DOI:** 10.1080/22221751.2024.2324415

**Published:** 2024-02-26

**Article title:** Robust neutralization assay based on SARS-CoV-2 S-protein-bearing vesicular stomatitis virus (VSV) pseudovirus and ACE2-overexpressing BHK21 cells

**Authors:** Xiong H., Wu Y., Cao J., Yang R., Liu Y., Ma J., Qiao X., Yao X., Zhang B., Zhang Y., Hou W., Shi Y., Xu J., Zhang L., Wang S., Fu B., Yang T., Ge S., Zhang J., Yuan Q., Huang B., Li Z., Zhang T., Xia N.

**Journal:** Emerging Microbes & Infections

**Bibliometrics:** Volume 09, Number 01, pages 2105-2113

**DOI:** 10.1080/22221751.2020.1815589

This article was originally published with errors in Figure 3A. The correct version of the Figure 3A is shown below:

**Figure 3.**
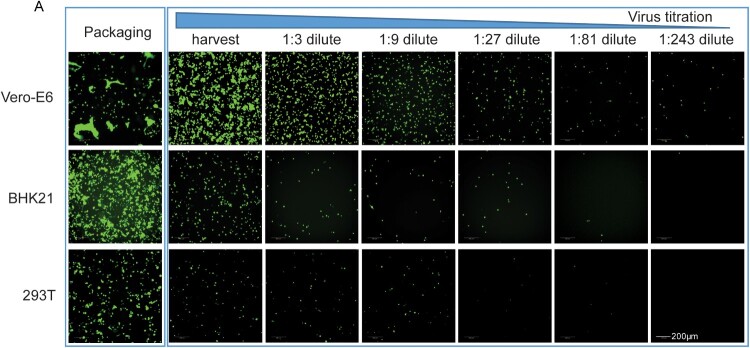
Comparison of the packaging efficiency of VSVdG-SARS-CoV-2-Sdel18 in various cell lines. Vero-E6, BHK21 and 293T cells were used to package the VSVdG-SARS-CoV-2-Sdel18 virus. (A) The left picture shows the cells used to package recombinant virus, recorded 48 h post infection with VSVdG-EGFP-G. The right figures show the infectivity of virus produced by three cell lines. The harvested virus was diluted and tested in BHK21-hACE2 cells.

